# Association Between Gut Microbiota and Autoimmune Thyroid Disease: A Systematic Review and Meta-Analysis

**DOI:** 10.3389/fendo.2021.774362

**Published:** 2021-11-17

**Authors:** Boshen Gong, Chuyuan Wang, Fanrui Meng, Haoyu Wang, Bo Song, Yang Yang, Zhongyan Shan

**Affiliations:** Department of Endocrinology and Metabolism, Institute of Endocrinology, National Health Commission (NHC) Key Laboratory of Diagnosis and Treatment of Thyroid Diseases, The First Affiliated Hospital of China Medical University, China Medical University, Shenyang, China

**Keywords:** autoimmune thyroid disease, gut microbiota, Hashimoto thyroiditis (HT), Graves’ disease (GD), meta-analysis

## Abstract

**Background:**

Autoimmune thyroid disease (AITD) is characterized by thyroid dysfunction and deficits in the autoimmune system. Growing attention has been paid toward the field of gut microbiota over the last few decades. Several recent studies have found that gut microbiota composition in patients with AITD has altered, but no studies have conducted systematic reviews on the association between gut microbiota and ATID.

**Methods:**

We searched PubMed, Web of Science, Embase, and Cochrane databases without language restrictions and conducted a systematic review and meta-analysis of eight studies, including 196 patients with AITD.

**Results:**

The meta-analysis showed that the alpha diversity and abundance of certain gut microbiota were changed in patients with AITD compared to the controls. Chao1,the index of the microflora richness, was increased in the Hashimoto’s thyroiditis group compared to controls (SMD, 0.68, 95%CI: 0.16 to 1.20), while it was decreased in the Graves’ disease group (SMD, -0.87, 95%CI: -1.46 to -0.28). In addition, we found that some beneficial bacteria like *Bifidobacterium* and *Lactobacillus* were decreased in the AITD group, and harmful microbiota like *Bacteroides fragilis* was significantly increased compared with the controls. Furthermore, the percentage of relevant abundance of other commensal bacteria such as *Bacteroidetes*, *Bacteroides*, and *Lachnospiraceae* was increased compared with the controls.

**Conclusions:**

This meta-analysis indicates an association between AITD and alteration of microbiota composition at the family, genus, and species levels.

**Systematic Review Registration:**

PROSPERO, identifier CRD42021251557.

## Introduction

Hashimoto’s thyroiditis (HT) and Graves’ disease (GD) are the main types of autoimmune thyroid disease (AITD). AITDs are the most common organ-specific autoimmune disorders. Although the clinical manifestations of GD and HT are different, such as hyperthyroidism and hypothyroidism, respectively, GD and HT share similar immune-mediated mechanisms of disease, even alternating from one to the other (1, 2). Many studies have revealed the possible causes of AITD, such as genetic susceptibility factors, dysregulation of the immune system, inflammation, stress, and other environmental factors; however, its etiology remains unclear (3, 4).

Emerging evidence suggests that the alterations of the gut microbiota play a key role in the development and progress of AITD in individuals. From the embryology aspect, the thyroid and gut share a common embryological origin, explaining some morphological and functional similarities between the gut and thyroid follicular cells (5). The association between autoimmune thyroid disorders and gut autoimmune disease atrophic gastritis was first described in the early 1960s (6). More recently, owing to the development of the 16S ribosomal RNA (16SrRNA) gene sequencing technique, the gut microbiota, which comprises trillions of microorganisms, has been proposed to be involved in the pathogenesis of many autoimmune diseases, such as type 1 diabetes, lupus nephritis, Rheumatoid arthritis, Celiac Disease, and AITD (7–10). Although there is no direct evidence that AITD and gut microbiota have a cause-effect relationship, several studies have suggested that the thyroid-gut axis has beneficial or detrimental effects on thyroid function (11). The gut microbiota shapes the thyroid mainly through the following possible microbial-related mechanisms. First, dysbiosis leads to the damaged intestinal barrier and increased intestinal permeability, allowing the antigens to pass into the circulation and activate the immune system (12). Second, the antibodies in the circulation may react with the bacterial antigen and enhance the activation of the inflammasome in the thyroid gland (13). Guo et al. (14) has found that the expression of the inflammasome, including the NOD-like receptor (NLR) family pyrin domain containing 3 (NLRP3), AIM2, caspase-1, and IL-1β mRNA and protein from patients with HT, was significantly increased, which can be regulated by the gut microbiota and its metabolism (15–17). Third, the short-chain fatty acids (SCFAs),metabolites of commensal bacteria fermentation of dietary fiber, are speculated to play a crucial role in the development, functioning, and modulation of the immune system (18, 19). For example, butyrate, a SCFAs, is associated with reduced levels of TNF-α, IL-6 and suppressed activation of the NLRP3 inflammasome *via* GPR109A (20).

Recently, many researchers have found that AITD patients have reduced α diversity and abundances of certain microbiota compared with healthy controls (21–23). The α diversity mainly contains community diversity (Simpson and Shannon) and community richness indices (ACE and Chao1) (24). Among the AITD patients, those with HT tend to have a higher Chao1 value than healthy volunteers; however, patients with GD have a lower Chao1 value than the controls (25, 26). In addition, the current results revealed the correlation between the clinical parameters of AITD, such as TRAb or TPOAb and the certain microbiota (22, 27, 28). For example, Chen et al. (29) found that the proportion of *Synergistetes* was negatively correlated with TRAb, and Jiang et al. (30) found that *Lactobacillus* was positively correlated with TRAb. At the phylum level, Yang et al. (31) found a higher *Firmicutes*/*Bacteroidetes* ratio in GD patients than in the control group, which may be relevant to inflammation disease, whereas Hanaa et al. (32) found that the ratio was significantly decreased in patients with AITD. Due to different conflicting data, a further study of the association between the gut microbiota and AITD is needed. To better understand the potential role of gut microbiota in the pathogenesis of AITD,we carried out a meta-analysis to assess the alteration in the microbial population between patients with AITD and healthy controls at different levels.

## Materials and Methods

### Search Strategy

We conducted a systematic literature search in PubMed, Web of Science, Embase, and Cochrane databases up to August 2021 using the following search string: (thyroiditis OR Hashimoto Disease OR Thyroiditis, Autoimmune OR Hashimoto Thyroiditis OR Thyroiditis, and Chronic Lymphocytic OR Chronic Lymphocytic Thyroiditis OR Thyroid Diseases OR Graves’ Disease OR Disease, Graves OR Goiter, Exophthalmic OR Basedow’s Disease OR Hyperthyroidism, Autoimmune) AND (microbiota OR Gut Microbiome OR Microbial Community OR Microbial Community OR Gastrointestinal Microbiome OR Microbiome OR Gut Flora OR Gastrointestinal Microbiota OR Microflora, Gastrointestinal OR Gastric Microbiome OR Intestinal Microbiota OR Intestinal Flora).

### Inclusion and Exclusion Criteria

Studies were considered eligible if they met the following criteria: 1) investigating the gut microbiota and patients diagnosed with AITD. 2) providing sufficient data on the relationship between AITD and intestinal microbiota and could be extracted to analyze the 95% confidence interval (CI). 3) written in English. 4) full-text availability. However, comments, animal model subjects, conference abstract, and reviews were excluded. We also excluded studies with incomplete outcome data on the percentages or relative abundance of gut microbiota and studies with fewer than 20 participants.

### Data Extraction

Three reviewers independently extracted the following data from each study: authors, publication year, country of population, population age, clinical parameters of thyroid function, diagnosis of AITD, and microbiology assessment methods.

### Quality Assessment

Two reviewers completed the quality assessment using the Newcastle-Ottawa scale (NOS) to evaluate all the included studies, comprising the trial group selection, comparability, and exposure. The total score ranged from 5−9, where a higher score represents a higher quality of assessment. All the discrepancies or poor agreement were resolved through a consensus discussion with a third author.

### Statistical Analysis

Standardized mean differences (SMD) were calculated between the HT and GD groups to assess the bacterial alpha diversity indices (Chao1). SMD >0 indicates that participants with HT have a higher level of richness in the intestinal microflora. A fixed- and random-effects were used to assess the percentages or relative abundances of certain gut microbiota with AITD compared with the healthy controls. We examined the statistical heterogeneity using the I2 statistic. I2 values of 25%, 50%, and 75% were related to low, moderate, and high heterogeneity, respectively. A Random-effects model was used to pool the results when high heterogeneity (I2>50%) existed. Additionally, the fixed-effects model was used if the heterogeneity was low. Furthermore, we performed a sensitivity analysis as well as Begg’s test to assess the potential influences of bias. All statistical analyses were performed using Stata software (version 12.0).

## Results

### Characteristics of Included Studies

**Figure 1:**
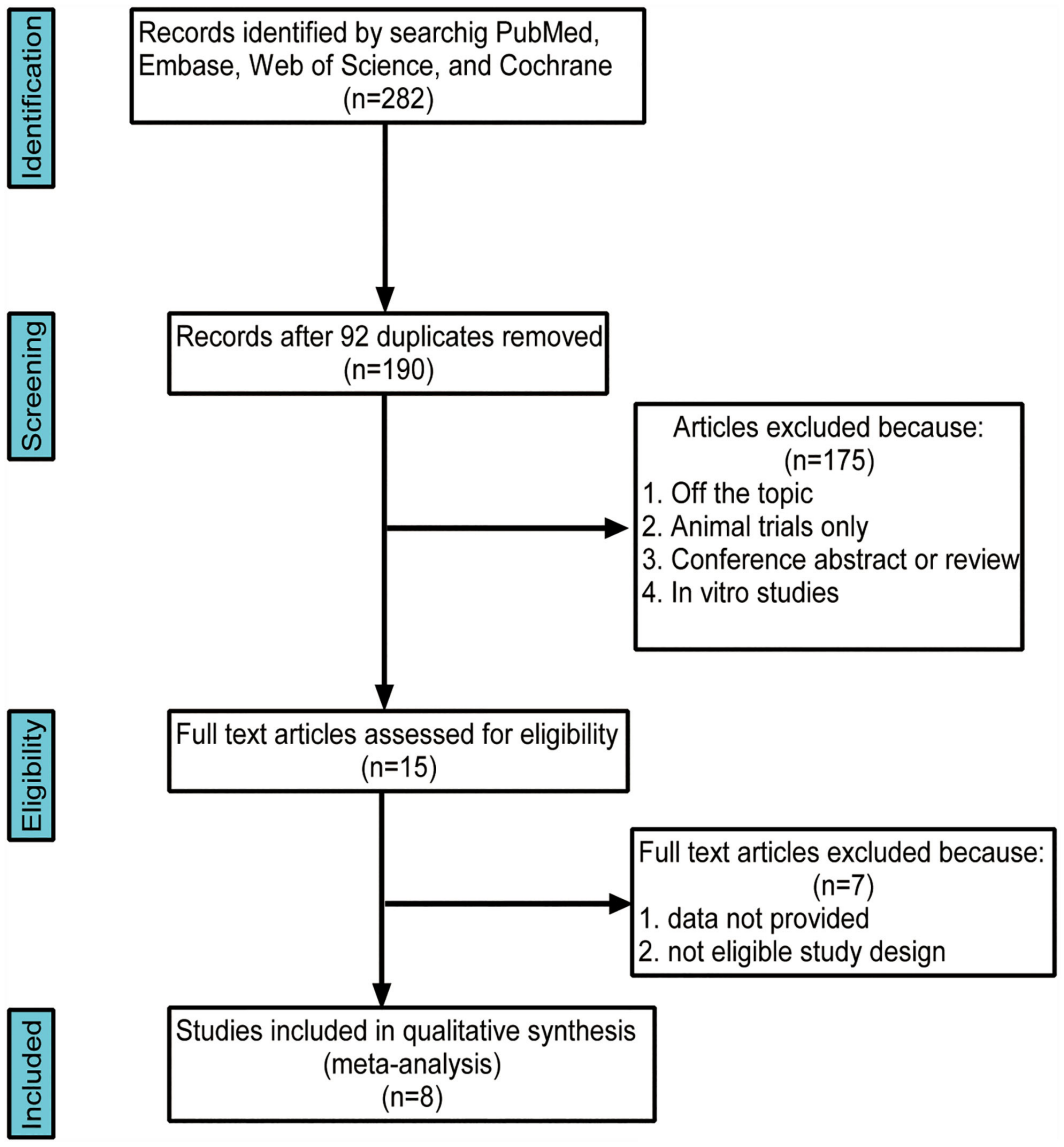
Flow chart of the search strategy and study selection progress.

The initial literature searches retrieved 282 records from the online database. Among them,92 studies were excluded for duplication, and after the review of titles and abstracts,175 articles were eliminated because they did not fulfill the inclusion criteria. Then, we evaluated the remaining 15 articles individually; seven were excluded because the studies did not provide quantitative or appropriate data on the gut microbiota relative abundance. Finally, eight studies were included in our meta-analysis **(** **Figure 1**). This meta-analysis included 196 patients with AITD and 160 age-matched healthy controls (**Table 1**). The clinical parameters of AITD and microbiology assessment methods used in the included studies are shown in **Table 2**. Fecal samples were collected, and microbiota was analyzed by pyrosequencing or high-throughput sequencing of the 16SrRNA gene, real-time PCR, and PCR-DGGE of the 16SrRNA gene (33). The percentage of gut microbiota composition at a different level and relevant abundance were analyzed based on the assessment methods, preventing the potential deviation caused by the detection methods.

**Table 1:** Characteristics of the studies included in the systematic review and meta-analysis.

Author	Country	AITD(n)	Age	Control(n)	Age(years)	Score
Zhao (21)	China	28	44.29±10.7	16	44.63±10.33	8
Zawawy (32)	Egypt	20	38.6±11.1	30	39.7±10.9	6
Ishaq (25)	China	29	40-60	12	40-60	7
Cornejo (28)	Spain	18	40.3±9.6	11	48.8±6.2	6
Ishaq (26)	China	27	35-50	11	35-50	8
Jiang (30)	China	45	16-65	59	22-71	6
Chen (29)	China	15	28.87±6.79	14	27.29±5.73	7
Zhou (33)	China	14	45-65	7	48-60	5

AITD, autoimmune thyroid disease.

**Table 2:** Clinical parameters and microbiology assessment of selected studies.

Author	Diagnosis of AITD	GenderM/F	Clinical parameters of thyroid	Sample	Microbiology assessment
Zhao	HT	3/25	TPOAb:460.23±341.38IUmlL	Fecal	High-throughput sequencing
			TG-Ab:353.47±345.29IU/mL		
			TSH:2.590.97μIU/ml		
			FT3:2.78±0.39pg/ml		
			FT4:1.06±0.14ng/ml		
Zawawy	HT	1/6	TPO-Ab:242±227.3IU/mL	Fecal	RT-PCR
			TSH:18.2±29.4μIU/ml		
			FT3:2.3±1 pg/ml		
			FT4:0.8±0.36 ng/ml		
	GD	9/4	TR-Ab:26.7±9.3IU/mL		
			TSH:0.009±0.001μIU/ml		
			FT3:20.7±7 pg/ml		
Ishaq	HT	9/20	TPO-Ab: >15IU/mL	Fecal	High-throughput sequencing, PCR-DGGE
			TSH: >5μIU/ml		
			T4: <4.2μg/dl		
			T3: <0.78 ng/ml		
Cornejo	HT	0/9	TPO-Ab:1186.7±358.4IU/mL	Fecal	High-throughput sequencing, RT-PCR
			FT3:3.8±0.2pmol/L		
			FT4:15.4±2.2pmol/L		
	GD	2/7	TSI-Ab:16.8±34.1 IU/mL		
			FT3:5.5±2.3		
			FT4:15.2±3.1		

Ishaq	GD	10/17	>3000U/mL (18/27)	Fecal	High-throughput sequencing, RT-PCR, DGGE
			TSH< 0.07μIU/ml(17/27)		
			FT3:13.77±1.40		
			FT4:76.43±10.47		
Jiang	GD	12/33	TPOAb:217.37±148.21IU/mL	Fecal	High-throughput sequencing
			TG-Ab:296.51±408.30IU/mL		
			TSH:0.04±0.11mIU/L		
			FT3:20.04±8.69		
			FT4:49.23±24.46		
Chen	GD	7/8	TPOAb:110.55±118.50IU/mL	Fecal	High-throughput sequencing
			TGAb:983.65±1717.64IU/mL		
			TR-Ab:6.62±5.39 IU/L		
			FT3:22.18±10.44 pmol/L		
			FT4:51.45±18.63 pmol/L		
Zhou	GD	5/9	Not mention	Fecal	PCR-DGGE, RT-PCR

RT-PCR, real time PCR; PCR-DGGE, PCR amplification for Denaturing Gradient Gel Electrophoresis; TPO-Ab, thyroid peroxidase antibody; TG-Ab, thyroglobulin antibodies; TR-Ab, thyrotropin receptor antibodies; HT, Hashimoto Thyroiditis; GD, Graves’ disease FT3, Free triiodothyronine; FT4, Free thyroxine; T3, Triiodothyronine; T4, Thyroxine.

### Alpha Diversity

**Figure 2:**
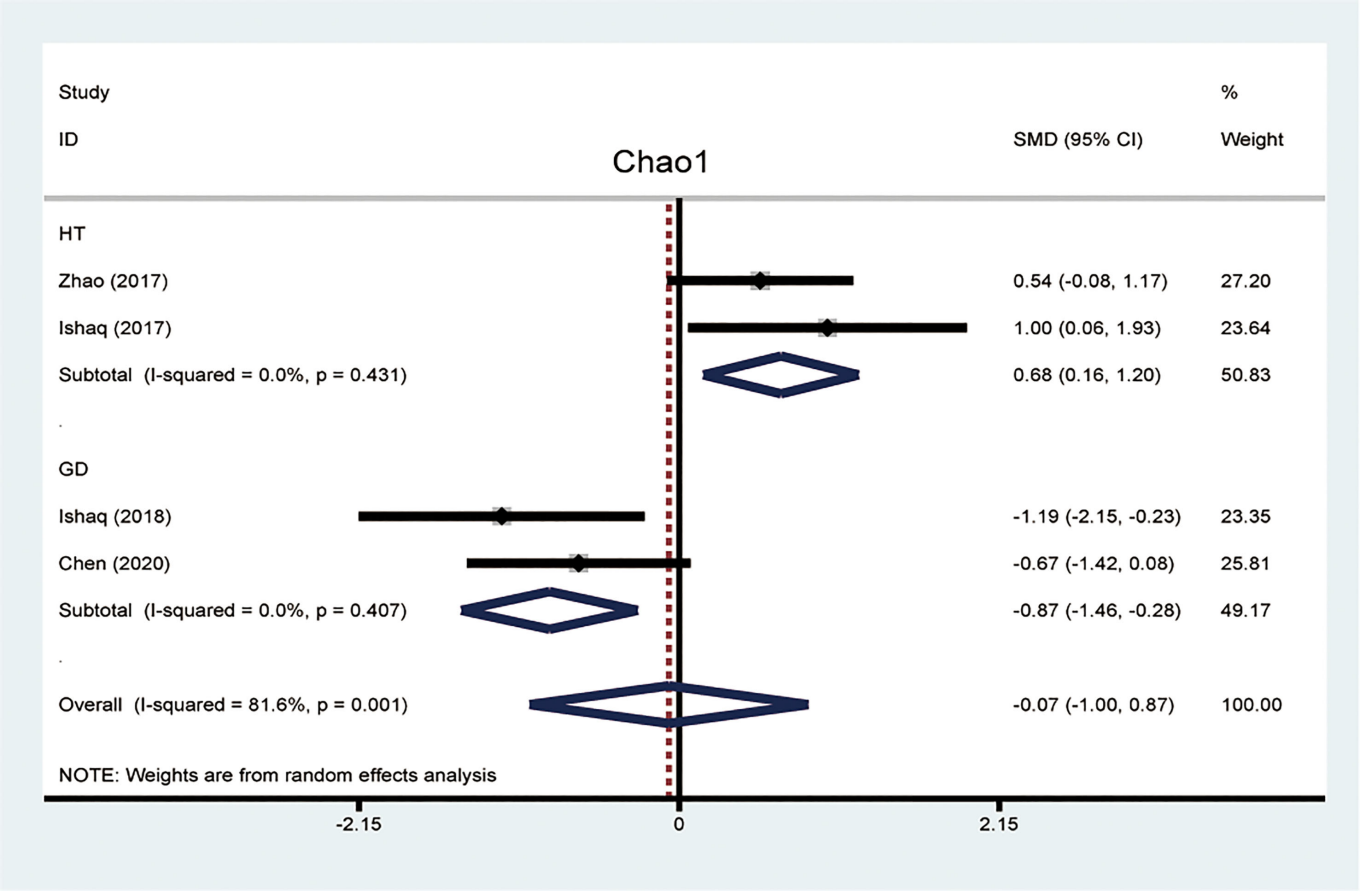
Forest plots of differences of Chao1 index between patients with Hashimoto thyroiditis (HT) and Graves’ disease (GD).

The alpha diversity was used to analyze the complexity of species diversity, including diversity indices (Simpson and Shannon) and richness indices (ACE and Chao1). The Chao1 index was a reflex of microflora richness. The meta-analysis of four studies reported data about the Chao1 index between patients with AITD and healthy controls. We found that the Chao1 index was significantly different between the HT and GD groups compared with the controls. To explore the source of heterogeneity, we divided these four articles into two subgroups based on the entities of AITD. The random-effects model of subgroup meta-analysis showed that the Chao1 index was increased in the HT group compared to the controls (SMD, 0.68, 95%CI: 0.16 to 1.20), while it was decreased in the GD group (SMD -0.87, 95%CI: -1.46 to -0.28). The effect size (z=0.14,P=0.886) was relatively small. There was no evidence of between-study heterogeneity (I2 = 0%; **Figure 2**).

### Phylum

#### Firmicutes

**Figure 3:**
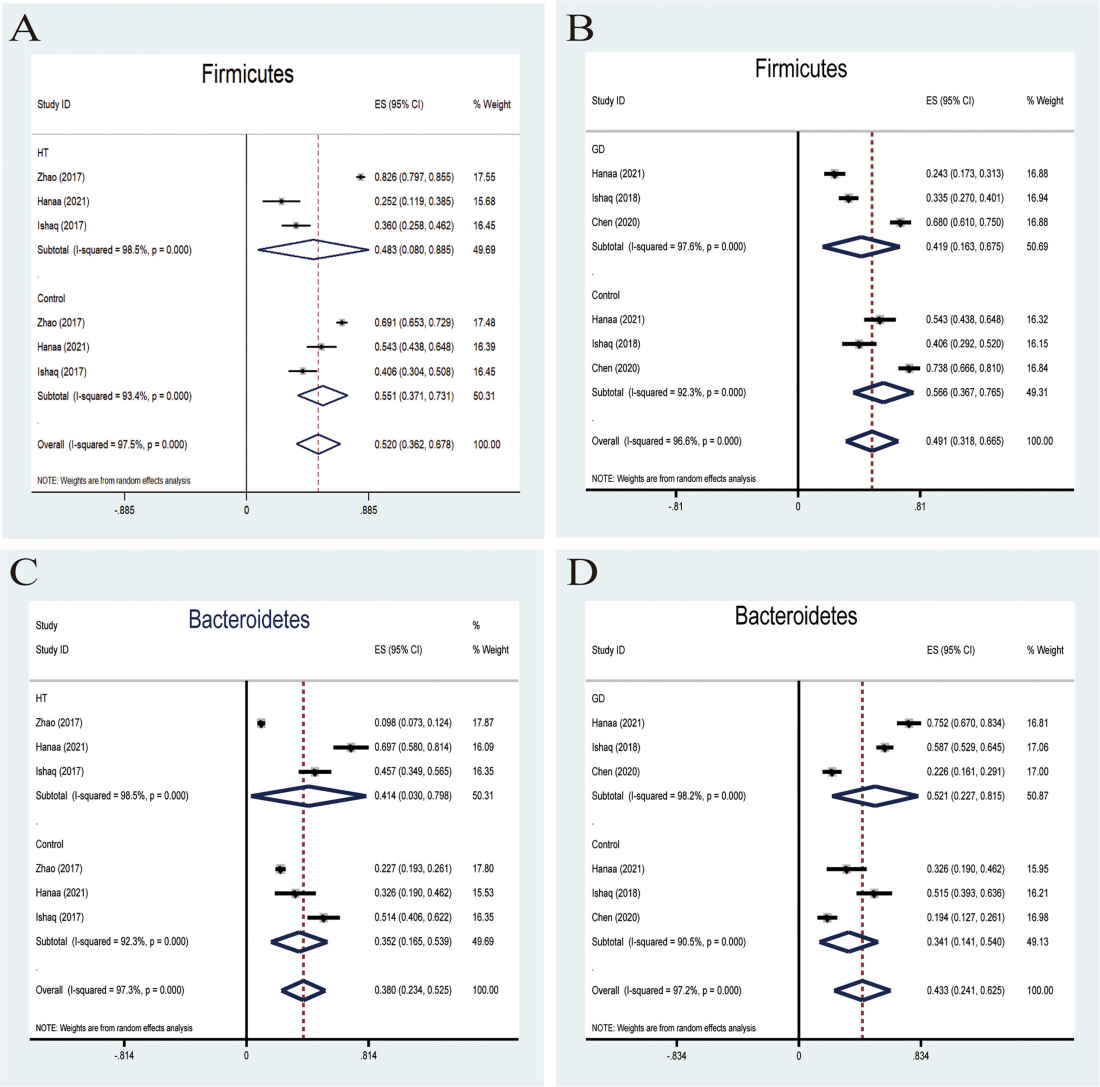
Forest plot of the percentages of *Firmicutes* and *Bacteroidetes* comparing people with Graves’ disease (GD) or Hashimoto Thyroiditis (HT) to healthy controls. **(A–D)** Random-effects models were used to assess the percentages of *Firmicutes* and *Bacteroidetes* in the detected intestinal microbiota, contributing to higher between-study heterogeneity (I2>50%).

*Firmicutes*, one of the predominant phyla of the human gut microbiota, showed a lower abundance in patients with AITD than the controls (34). A random-effects model showed that the percentage of *Firmicutes* in the detected sample of HT was 48.3% (95%CI: 0.080 to 0.885) and 55.1% (95%CI: 0.371 to 0.731; **Figure 3A**) in the controls. The effect size (Z=6.45, P=0.000) was significant. While it was 41.9% (95%CI: 0.163 to 0675) in the GD group and 56.6% (95%CI: 0.367 to 0.765; **Figure 3B**) in the controls. The effect size (Z=5.55, P=0.000) was large and significant. The ratio of bacterial percentage between the HT group (48.3%) and the control group (55.1%) was 0.88 (**Figure 6A**),and that of the GD (41.9%) group and the controls (56.5%) was 0.74 (**Figure 6B**).

#### Bacteroidetes

Six studies were used to evaluate the percentage of *Bacteroidetes*. A random-effects meta-analysis model showed that the percentage of *Bacteroidetes* of the HT group was 41.4% (95%CI: 0.030−0.798), slightly higher than that in the control group 35.2% (95%CI: 0.165 to 0.539; **Figure 3C**). Its effect size (Z=5.78, P=0.000) was significant. The same meta-analysis model was used in the GD group; the percentage of *Bacteroidetes* of the GD group was 52.1% (95%CI: 0.227 to 0.815), while the control group was 34.1% (95%CI: 0.141 to 0.540; **Figure 3D**). The effect size (Z=5.29, P=0.000) was significant and large. The ratio of bacterial percentage between the HT group (41.4%) and the control group (35.2%) was 1.13 (**Figure 6A**), and between the GD group (52.1%) and the control group (34.1%), it was 1.53 (**Figure 6B**).

### Genus

#### *Bifidobacterium* and *Lactobacillus*

**Figure 4:**
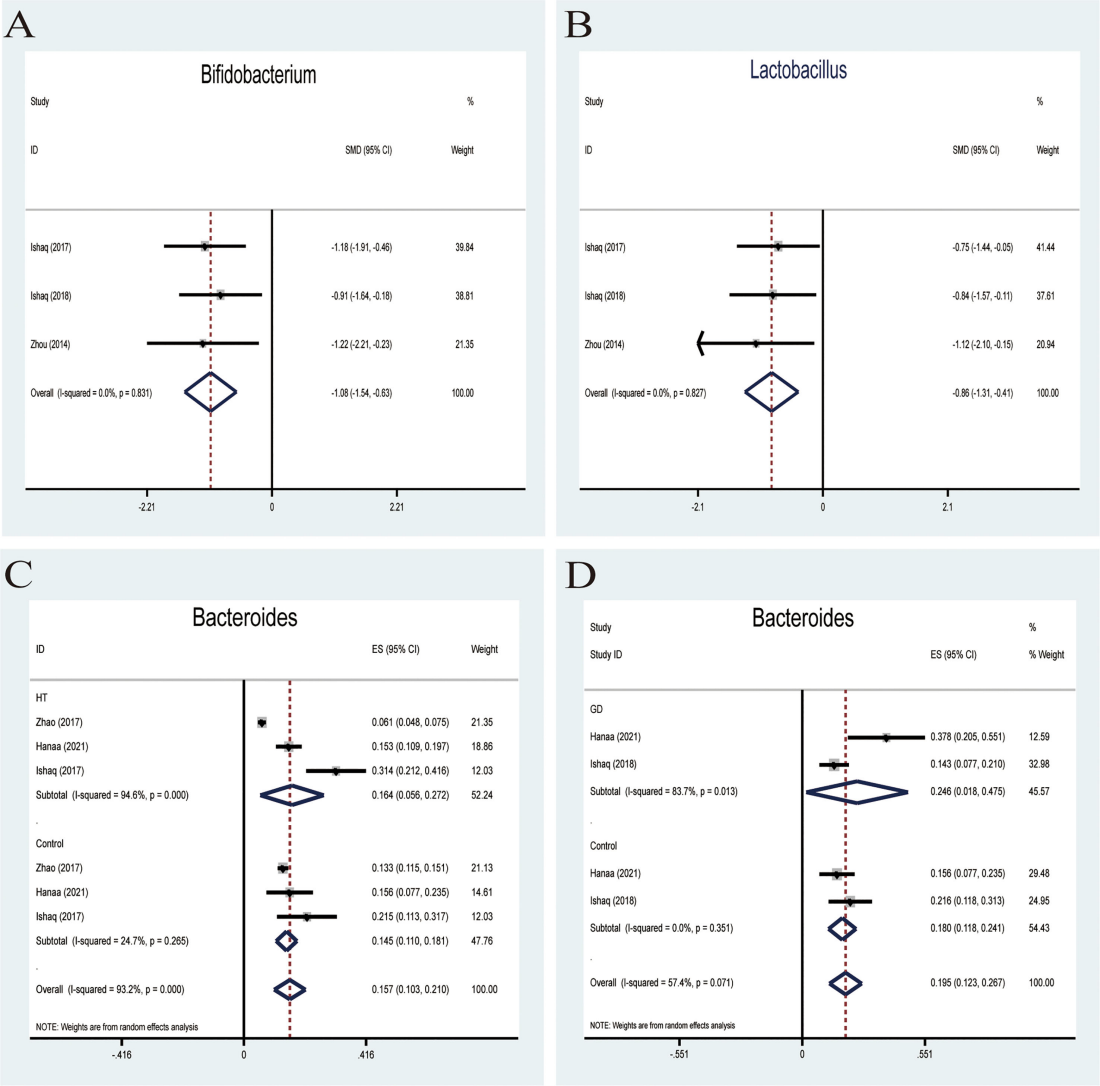
Forest plot of the relative abundance of *Bifidobacterium* and *Lactobacillus* and percentages of *Bacteroides in* autoimmune thyroid disease (AITD). **(A, B)** Fixed-effects models were used to analyze the relative abundance of *Bifidobacterium* and *Lactobacillus*, comparing patients with AITD to healthy controls. **(C, D)** Random-effects models were used to assess the percentages of *Bacteroides*, contributing to higher between-study heterogeneity (I2>50%).

Probiotics belonging to *Bifidobacterium* and *lactobacillus* have been exploited and invented for their benefits in treating many pathological conditions (35). The effectiveness of these two main probiotic strains is used for treatment or therapeutic purposes. A fixed-effects meta-analysis indicated a lower relative abundance of *Bifidobacterium* in patients with AITD than the controls (SMD -1.08,95%CI: -1.54 to -0.63; **Figure 4A**). The effect size (Z=4.66, P=0.000) was significant. Besides, a fixed-effects model also showed that the relative abundance of *lactobacillus* decreased compared to that in healthy controls (SMD -0.86,95%CI: -1.31 to -0.41; **Figure 4B**). Its effect size (Z=3.78, P=0.000) was significant and moderate.

#### Bacteroides

*Bacteroides* is a pro-inflammatory bacterium that contributes to the pathogenesis of inflammatory bowel disease (36). Five studies analyzed the percentage of *Bacteroides* in patients with AITD. A random-effects meta-analysis showed 16.4% of *Bacteroides* in the detected sample of patients with HT (95%CI: 0.056 to 0.272), while the percentage in healthy control was 14.5% (95%CI: 0.110 to 0.181; **Figure 4C**). The effect size (Z=5.78, P=0.000) was significant. The percentage of *Bacteroides* in the GD group was 24.6% (95%CI: 0.163 to 0.675) and 18% (95%CI: 0.118 to 0.241; **Figure 4D**) in the healthy control group. Its effect size (Z=5.29,p=0.000) was significant and large. The ratio of bacterial percentage between the HT group (16.4%) and the control group (14.5%) was 1.13 (**Figure 6A**). In contrast,it was 1.37 between the GD group (24.6%) and the control group (18.0%) (**Figure 6B**).

### Family and Species

#### Lachnospiraceae

**Figure 5:**
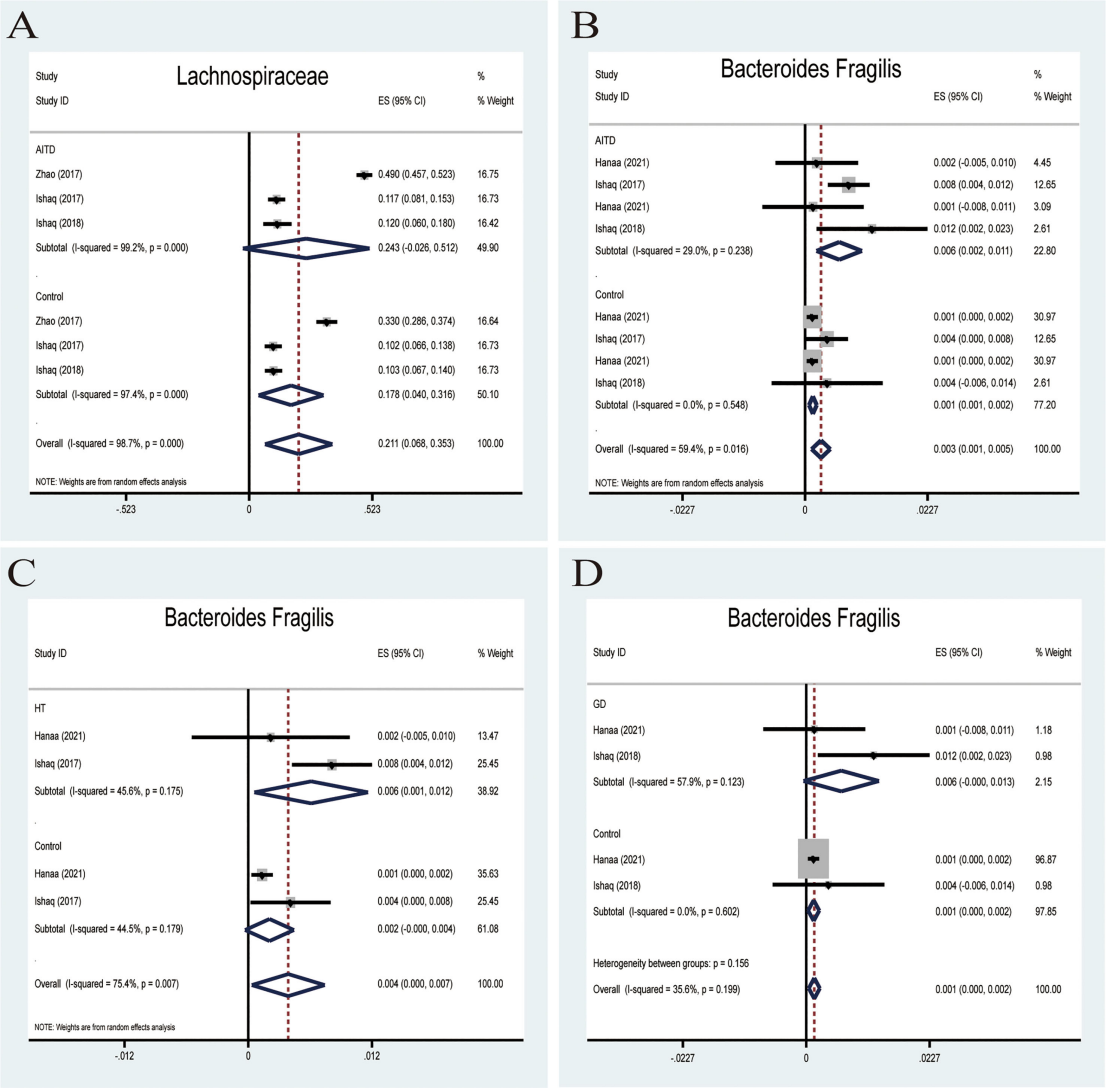
Forest plot of the percentages of *Lachnospiraceae and Bacteroides Fragilis* in autoimmune thyroid disease (AITD). **(A)** A random-effects model was used to assess the percentages of *Lachnospiraceae*, contributing to higher between-study heterogeneity (I2>50%). **(B)** A fixed-effects model was used to assess *Bacteroides Fragilis*, comparing patients with AITD to healthy controls. **(C, D)** Random-effects models were used to assess the percentages of *Bacteroides Fragilis* comparing people with Graves’ disease (GD) or Hashimoto Thyroiditis (HT) to healthy controls.

**Figure 6:**
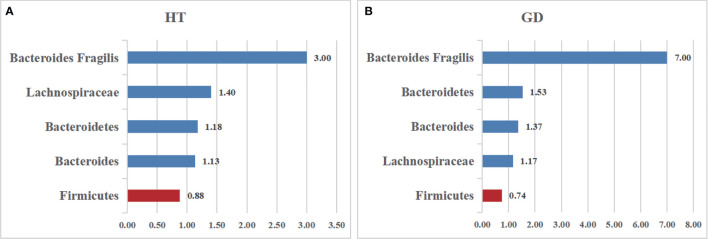
The relative abundance of the included bacteria in our meta-analysis. **(A)** The ratio of the gut microbiota percentages in patients with HT. **(B)** The ratio of the bacteria percentages of patients with GD. A value greater than 1 shows a higher abundance of microbiota (*Bacteroides Fragilis*, *Lachnospiraceae*, *Bacteroidetes*, *Bacteroides*), whereas a value less than 1 indicates lower abundance compared to the healthy controls. (*Firmicutes*).

*Lachnospiraceae*, a member of the core gut microbiota, colonizes the host’s intestinal tract from birth. Besides, members of *Lachnospiraceae* are the main microbiota producing SCFA, regulating the inflammasome activation (37). A random-effects meta-analysis showed that the percentage of *Lachnospiraceae* in the detected sample was 24.3% (95%CI: -0.026 to 0.512), whereas that in healthy controls was 17.8% (95%CI: 0.040 to 0.316; **Figure 5A**). The effect size (Z=2.89,P=0.004) was significant. The ratio of bacterial percentage between HT (30.3%) and the control group (21.6%) was 1.40 (**Figure 6A**), while between GD (12.0%) and the control group (10.3%), it was 1.17 (**Figure 6B**).

#### Bacteroides fragilis

*Bacteroides fragilis* was previously reported to stimulate the host immune system by upregulating NLRP3, IL-1β,IL-18,and caspase-1 mRNA and protein expression in EGCs and reducing the NLRP6 mRNA expression (38). All four studies reported that *B. fragilis* in the AITD group was significantly higher than that in the controls. A random-effects meta-analysis showed that the percentage of *B. fragilis* in the detected fecal sample was 0.6% (95%CI: 0.002−0.011) in patients with AITD and 0.1% (95%CI: 0.001−0.002) in healthy controls. The heterogeneity within the AITD group was low (29.0%; **Figure 5B**). Its effect size (Z=3.34, P=0.001) was significant. Additionally, the fixed-effects meta-analysis showed that the percentage of *Bacteroides fragilis of* patients with HT was 0.6% (95%CI: 0.001 to 0.012) compared to 0.4% in control group (95%CI: 0.000 to 0.007; **Figure 5C**). The effect size (Z=2.15, P=0.031) was significant. While the percentage of *Bacteroides fragilis* of patients with GD was 0.6% (95%CI: 0.000 to 0.013),higher than the healthy controls (0.1%; 95%CI: 0.000 to 0.002; **Figure 5D**). Its effect size (Z=2.85, P=0.004) was significant.

### Correlation With Clinical Parameters

Several studies have revealed the significant correlations between clinical thyroid-related antibodies like TPO-Ab and TR-Ab, and different bacterial abundances by Spearman’s correlation distance. The Spearman correlation coefficient R-value between microbiota and immune indices, including TPO-Ab and TR-Ab, demonstrated that certain microbiota significantly correlated with thyroid function tests (**Table 3**). Among all, the relative abundance of *Bacteroides* (R = -0.342, P = 0.014), *Dorea* (R = -0.341,P = 0.022), *Faecalibacterium* (R = -0.453, P = 0.014), *Synergistetes* (R = -0.711, P = 0.001),and *Coprococcus* (R = -0.499,P = 0.03) were negatively correlated with TPO-Ab or TR-Ab. In contrast, other commercial bacteria like *Blautia*, *Lactobacillus*, *Alistipes*, *Ruminococcaceae*, and *Enterobacteriaceae* were positively correlated with TPO-Ab.

**Table 3:** Potential correlation relationship between thyroid-related antibodies and gut microbiota.

Study	Family/Genus level	TPOAb		TRAb	
		r	P	r	P
Jiang,2020	Blautia	0.365	0.014	—	—
	Bacteroides	-0.342	0.021	—	—
	Lactobacillus	0.156	>0.05	0.092	>0.05
	Dorea	-0.341	0.022	—	—
Cornejo,2020	Alistipes	0.432	0.019	—	—
	Faecalibacterium	-0.453	0.014	—	—
	Ruminococcaceae	0.408	0.028	—	—
	Enterobacteraceae	0.416	0.025	—	—
Chen,2020	Synergistetes	-0.711	0.001	-0.702	0.000
	Lactobacillus	0.607	0.006	0.489	0.024
	Corprococus	-0.499	0.030	-0.366	0.103
	Phascolarctobacterium	-0.336	0.160	-0.544	0.011

TPO-Ab, thyroid peroxidase antibody; TR-Ab, thyrotropin receptor antibodies.

## Discussion

To the best of our knowledge, this is the first meta-analysis to demonstrate the association between gut microbiota and AITD. This association has become a very popular topic and field in recent years. Many researchers want to illuminate the association and underlying mechanism linking AITD and microbiota, expecting to discover a novel target for early diagnosis or reversal of the host’s disordered immune system through fecal microbiota transplantation. Owing to the development of 16SrRNA sequencing technologies, significant alterations of the abundance and composition of gut microbiota have been found between AITD and healthy controls, suggesting that the gut-thyroid axis may play a pivotal role in the development and progress of AITD (11).

Though HT and GD are considered the most common two forms of AITD, the pathogenesis and hallmark of HT and GD are quite different. The hallmark of HT is the high rate of TPO-Ab, and TG-Ab, while high TR-Ab level was detected in patients with GD (39). Besides, the alteration of certain gut microbiota compositions may be different between HT and GD. For example, the species community richness index Chao1 was significantly elevated in samples from patients with HT while reduced in patients with GD. Species richness is an important feature of ecological composition and community structure. The opposite indicates that HT patients have greater gut microbiota richness, which may be related to the bacterial overgrowth in the intestinal tract. In contrast, the GD patients may have less community abundance.

AITD has been linked to gut microbiota dysbiosis by different mechanisms, such as bacterial overgrowth, overactivation of the inflammasome, increased intestinal permeability, alteration of microbiota metabolites, and immune homeostasis (40). However, the mechanisms of the complex gut-thyroid axis have not been fully elucidated. *Firmicutes* and *Bacteroidetes* are the main dominant microbiota at the phyla level. Traditionally, the ratio of *Firmicutes* and *Bacteroidetes* has been implicated in the predisposition of disease conditions. In our meta-analysis, the rate of *Firmicutes* and *Bacteroidetes* ratio of patients with AITD showed a lower level than their healthy counterparts. Besides, AITD patients showed a notable alteration in gut microbiota composition as compared to the controls. A random-effect model indicates that people with AITD showed a significantly increased relative abundance of pathogenic bacteria and a decreased proportion of beneficial bacteria such as *Lactobacillus* and *Bifidobacterium*. *Lactobacillus* and Bifidobacterium could be used as a probiotic to modulate the immune response and have no adverse effect on developing an experimental autoimmune thyroiditis mice model (41). In addition, *Lactobacillus* has been proved to protect TH17 cells and support barrier integrity by secreting IL-22 and IL-17. The Th17/Treg imbalance may cause inflammatory disorders, indicating that *Lactobacillus* participates in immune system balance. *Overall*, *Bifidobacterium* and *Lactobacillus* showed anti-inflammatory effects and shield our body from pathogen. What’s more the Increased *B. fragilis* species may account for the upregulation of IL-18, IL-1β, and caspase-1, promoting the inflammatory response. Besides, *B. fragilis* can active the expression of NLRP3,which have been found overexpression in HT patients. However, the interaction between gut microbiota and inflammasome was still unclear. Another reasonable hypothesis of the role that the microbiota plays in the progress of AITD is molecular mimicry. The Antigenic properties of proteins of certain intestinal bacteria may bind TPO-Ab and TG-Ab, which are the main AITD’s clinical diagnostic parameters (42).

Accumulating evidence suggests that the gut microbiota can regulate the local intestinal immune system and moderate the immune system outside the gastrointestinal system by metabolites secretion. The immunological system resulting in the development of AITD is potentially correlated with microbial metabolism. SCFAs, mainly butyrate, acetate, and propionate, are primarily produced by *Bacteroidetes*, *Bifidobacterium*, *Faecalibacterium*, and *Enterobacteria*, which are rich sources of energy for the host (43). Butyrate is one of the most important metabolites produced by butyrate-producing bacteria such as *Firmicutes*, enhancing the intestinal barrier and mucosal immunity. Thus, the decreased phylum *Firmicutes* in the AITD group may account for the increased intestinal permeability. Studies found a notable increase in the expression of inflammasome components, including NLRP1,NLRP3,NLRC4,and AIM2 in HT patients, which can be activated by intestinal microbiota through PAMPs (pathogen-associated molecular pattern molecules (PAMPs) or DAMPs (damage-associated molecular pattern molecules (DAMPs) patterns. Notably, the IL-1β, the subsequent effector molecules downstream of inflammasomes, can modulate the microbiota by regulating the production of antimicrobial peptides (AMPs) (15). In addition, trimethylamine N-oxide (TMAO), a gut microbiota-dependent product, has been found to participate in the progression of vascular calcification and endothelial dysfunction by enhancing the activation and formation of the NLRP3 inflammasome, ASC, IL-1β, and caspase-1 (44). A study including a 1621 Czech population also supports the connection between *Helicobacter pylori* and HT. *H. pylori*, a gram-negative bacterium of the human microbiota, predominantly promotes the NLRP3 inflammasome and caspase-1 activation as well as IL-1β secretion *via* TLR4,MyD88,and NF-kB (45, 46). Moreover, certain microbiota may increase the permeability, allowing the toxins, antigens, or SCFA to pass into the blood circulation by activating the inflammatory system.

Furthermore, several correlation studies were performed to establish a relationship between thyroid autoimmunity parameters and specific bacteria based on Spearman correlation distance analysis. Spearman correlation analysis is one of the most common analysis to reveal the correlations between altered microbiota and various clinical parameters. In our study, the association between gut microbiota and AITD has already been explored, including the percent of certain microbiota increased or decreased compared to healthy controls. However, the altered abundance of gut microbiota may not indicate a direct association between gut microbiota and AITD. To bridge this gap, the correlation between specific microbiota and two clinical parameters (TPOAb and TRAb) of thyroid may provide guidance for the future study or diagnosis of AITD. We found that some intestinal microbiota, such as *Ruminococcaceae*, *Blautia*, and *Alistipes*, were significantly positively correlated with TPO-Ab. In contrast, other bacteria such as *Bacteroides* and *Synergistetes* were negatively correlated with TPO-Ab or TR-Ab. Furthermore, the gut microbiota is correlated with cell functions such as membrane transport, genetic information, or other cellular processes (30). Notably, the increased level of antibodies and thyroid hormone in AITD patients might affect and change the composition and amount of intestinal microbiota (47, 48). Therefore, we hypothesized that the AITD might be associated with the alterations of the gut microbiota.

### Limitation of the Study

Several limitations of this meta-analysis should be considered. First, most studies did not consider the effect of dietary habits. The consumption of various food or fiber can influence the composition of the commensal microbiota and its metabolites, SCFA (47). Second, many of the included observational studies did not evaluate thyroid hormone influences and thyroid autoantibodies on gut microbiota composition. Third, seasonal variations influence gut microbiota composition. For example, the relative abundance of certain microbiota may be affected by the time of stool sample collection; however, only one study mentioned that stool samples were collected in Autumn. Fourth, a series of metabolism changes of AITD could also altered the composition of gut microbiota, thus, the results can only reflect an association between gut microbiota and autoimmune thyroid disease but not a causation relationship.

## Conclusion

This meta-analysis shows the interactions between the gut microbiota and the pathogenesis of AITD. Furthermore, certain beneficial intestinal bacteria decrease, such as *Bifidobacterium* and *Lactobacillus*, may be associated with the development of AITD to some extent. However, the way how microbiome affects the progress of AITD is still controversial, and the research data is limited. Therefore, a further multicenter approach is needed to clarify the underlying pathogenesis and progress of intestinal microbiota dysbiosis in AITD.

## Data Availability Statement

The original contributions presented in the study are included in the article/supplementary material. Further inquiries can be directed to the corresponding author.

## Author Contributions

BG and ZS conceived the study. CW, FM, HW, and YY participated in the statistical analysis. BG and BS drafted the article. All authors read and approved the final version of the manuscript.

## Funding

This study was supported by the Department of Education in Liaoning Province, China (LJKZ0742).

## Conflict of Interest

The authors declare that the research was conducted in the absence of any commercial or financial relationships that could be construed as a potential conflict of interest.

## Publisher’s Note

All claims expressed in this article are solely those of the authors and do not necessarily represent those of their affiliated organizations, or those of the publisher, the editors and the reviewers. Any product that may be evaluated in this article, or claim that may be made by its manufacturer, is not guaranteed or endorsed by the publisher.
